# The Risk of Vestibular Disorders with Semaglutide and Tirzepatide: Findings from a Large Real-World Cohort

**DOI:** 10.3390/biomedicines13051049

**Published:** 2025-04-26

**Authors:** Eman A. Toraih, Awwad Alenezy, Mohammad H. Hussein, Shahmeer Hashmat, Saitej Mummadi, Nawaf Farhan Alrawili, Ahmed Abdelmaksoud, Manal S. Fawzy

**Affiliations:** 1 Department of Surgery, School of Medicine, Tulane University, New Orleans, LA 70112, USA; etoraih@tulane.edu; 2 Department of Cardiovascular Perfusion, Interprofessional Research, College of Health Professions, Upstate Medical University, New York, NY 13210, USA; 3 Medical Genetics Unit, Department of Histology and Cell Biology, Suez Canal University, Ismailia 41522, Egypt; 4 Department of Family and Community Medicine, Faculty of Medicine, Northern Border University, Arar 91431, Saudi Arabia; dr.awwad@hotmail.com; 5 Ochsner Clinic Foundation, New Orleans, LA 70121, USA; mohamed.hussein@ochsner.org; 6 School of Medicine, Tulane University, New Orleans, LA 70112, USA; shashmat@tulane.edu (S.H.); smummadi@tulane.edu (S.M.); 7 Department of Internal Medicine, College of Medicine, Northern Border University, Arar 91431, Saudi Arabia; nawaf.alruwaili@nbu.edu.sa; 8 Department of Internal Medicine, University of California, Riverside, CA 92521, USA; aabelma@medsch.ucr.edu; 9 Center for Health Research, Northern Border University, Arar 73213, Saudi Arabia

**Keywords:** GLP-1 receptor agonists, semaglutide, tirzepatide, vestibular disorders, dizziness, vertigo, adverse effects, pharmacovigilance, type 2 diabetes, obesity

## Abstract

**Background/Objectives**: Glucagon-like peptide-1 receptor agonists (GLP-1RAs) have revolutionized the treatment of type 2 diabetes and obesity. While their metabolic benefits are well-established, their potential effects on vestibular function remain unexplored. This study investigated the association between GLP-1RA use and the risk of vestibular disorders. **Methods**: Using the TriNetX research network (accessed 3 November 2024), we conducted a retrospective cohort study of adults prescribed semaglutide (*n* = 419,497) or tirzepatide (*n* = 77,259) between January 2018 and October 2024. Cases were matched 1:1 with controls using propensity scores based on demographics and comorbidities. The primary outcome was new-onset vestibular disorders, analyzed at 6 months, 1 year, and 3 years after treatment initiation. **Results**: Both medications were associated with an increased risk of vestibular disorders. Semaglutide users showed a higher cumulative incidence (0.12% at 6 months to 0.41% at 3 years) compared to controls (0.03% to 0.16%, *p* < 0.001), with hazard ratios ranging from 4.02 (95% CI: 3.33–4.86) at 6 months to 4.95 (95% CI: 4.51–5.43) at 3 years. Tirzepatide users demonstrated similar patterns but lower absolute rates (0.10% at 6 months to 0.19% at 3 years vs. controls 0.04% to 0.15%), with hazard ratios from 3.19 (95% CI: 2.11–4.81) to 4.55 (95% CI: 3.43–6.03). The direct comparison showed a higher risk with semaglutide versus tirzepatide (RR 1.53–2.04, *p* < 0.001). **Conclusions**: GLP-1RA therapy is associated with an increased risk of vestibular disorders, with a higher risk observed with semaglutide compared to tirzepatide. These findings suggest the need for vestibular symptom monitoring in patients receiving these medications and warrant further investigation into underlying mechanisms.

## 1. Introduction

**Figure 1:**
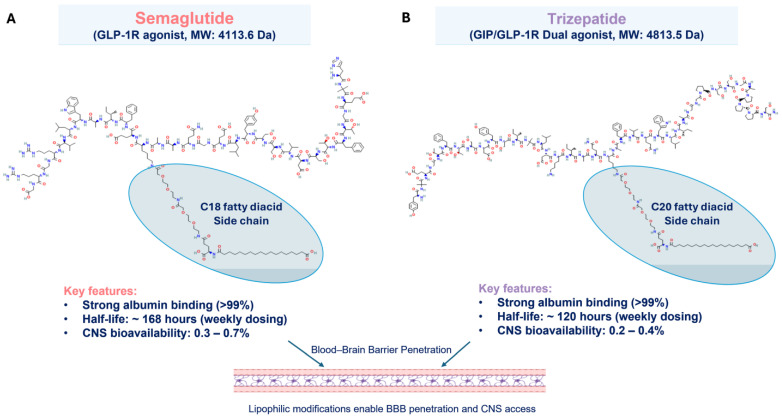
The chemical structure of Glucagon-like peptide-1 receptor agonists (GLP-1RA) and their key features. (**A**) Semaglutide and (**B**) tirzepatide. Both proteins have a long hydrocarbon chain (ellipse shape) attached to the main polypeptides, which facilitates passing through the blood–brain barrier (BBB). The chemical structure and data were obtained from the free source PubChem (Available online: https://pubchem.ncbi.nlm.nih.gov) (accessed on 15 April 2025).

Glucagon-like peptide-1 receptor agonists (GLP-1RAs), such as semaglutide (Drug Bank ID: DB13928, Chemical formula: C_187_H_291_N_45_O_59_) and tirzepatide (DB15171, C_225_H_348_N_48_O_68_), have become transformative agents in the management of type 2 diabetes mellitus (T2DM) and obesity due to their demonstrated benefits in glycemic control, weight loss, and potential cardiovascular protection [1,2,3,4]. Semaglutide and tirzepatide are structurally engineered to enhance pharmacokinetic properties. Semaglutide features a C18 fatty diacid chain conjugated to lysine at position 26 via a hydrophilic linker, while tirzepatide incorporates a C20 fatty acid moiety at lysine 20 through γ-glutamate and PEG spacers [5,6]. These lipidation modifications enable albumin binding, prolonging half-life (semaglutide: ~7 days; tirzepatide: ~5 days) and facilitating blood–brain barrier penetration [7] (Figure 1). This structural characteristic could be particularly relevant to their potential vestibular effects, as BBB permeability allows interaction with GLP-1 receptors in central balance-regulating regions like the vestibular nuclei complex [8]. By simulating the incretin hormone GLP-1, these medicines stimulate insulin release, lower glucagon, and slow stomach emptying, hence increasing fullness and reducing food intake [9].

Despite their metabolic advantages, growing questions concerning the wider physiological effects of GLP-1RAs demand cautious research, especially in relation to their impact on central nervous system (CNS) pathways linked to balance and spatial orientation [10,11]. Recent studies have documented otologic adverse events associated with GLP-1RA use, including vertigo, dizziness, and eustachian tube dysfunction [12]. For example, a systematic review of FDA adverse event reports revealed that vertigo was reported in 203 cases among GLP-1RA users, with semaglutide accounting for 1.17% of otologic adverse events [12]. Although vertigo is not commonly listed as a side effect in clinical trials, post-marketing surveillance data suggest a possible association that warrants further investigation [13,14].

Several potential mechanisms could support the hypothesis that GLP-1RA use could predispose to vestibular dysfunction: (1) through central vestibular modulation, as GLP-1Rs are densely expressed in brainstem nuclei (e.g., vestibular nuclei complex) and cerebellar regions that integrate sensory inputs for postural control. Preclinical studies demonstrate that GLP-1R activation alters neuronal excitability in these areas, potentially disrupting vestibulo-ocular reflex calibration and spatial navigation [15,16]. (2) GLP-1RAs induce nitric oxide-mediated vasodilation [17], which may perturb the delicate microcirculation of the stria vascularis, a structure that is vital for maintaining endolymphatic potential in the semicircular canals [18]. This type of disruption could mimic Ménière’s disease-like symptoms, including vertigo and imbalance [19]. (3) By modulating baroreflex sensitivity via GLP-1 receptors in the nucleus tractus solitarius, these agents may impair compensatory hemodynamic responses to postural changes, increasing susceptibility to orthostatic dizziness [20].

Vestibular disorders, including vertigo and benign paroxysmal positional vertigo, significantly impact the quality of life through balance disturbances and fall risk [21]. Although GLP-1 receptors are not conclusively identified in vestibular organs, their central expression patterns and systemic effects (e.g., blood pressure variability and fluid shifts) provide a plausible pathway for iatrogenic dysfunction [22,23]. Furthermore, there have been increasing anecdotal reports of dizziness and balance disturbances among GLP-1RA users, highlighting the need for a systematic investigation of this potential association [24].

GLP-1RAs have seen a significant increase in popularity, with prescription rates rising by 221.0% between 2016 and 2021 [25]. As their use expands across diverse patient populations, it is crucial to thoroughly assess their safety profile, including potential risks to vestibular health. To date, no comprehensive studies have directly examined the association between GLP-1RA therapy and vestibular disorders. Utilizing the extensive longitudinal dataset from the TriNetX network, our study aims to address this critical knowledge gap by investigating the incidence of vestibular disorders among GLP-1RA users. The findings will provide valuable insights to inform clinical monitoring and safety considerations for these widely prescribed medications.

## 2. Materials and Methods

### 2.1. Data Source and Study Design

This retrospective cohort study utilized data from the TriNetX research network, a global federated database containing anonymized electronic health records from various healthcare organizations (accessed on 3 November 2024). We analyzed real-world data from 1 January 2018 to 31 October 2024, leveraging TriNetX’s longitudinal data capabilities to track patient diagnoses, treatments, and outcomes. The study design and analysis adhered to ethical guidelines for the secondary use of health data, ensuring complete data anonymization and regulatory compliance.

### 2.2. Study Population and Eligibility Criteria

We identified adults aged ≥18 years with diabetes or obesity who were newly prescribed either semaglutide or tirzepatide. The study period encompassed multiple FDA approvals: semaglutide for T2DM (Ozempic, December 2017) and chronic weight management (Wegovy, June 2021), and tirzepatide for T2DM (Mounjaro, May 2022) and chronic weight management (Zepbound, November 2023). Patients with pre-existing vestibular disorders (ICD-10 code H81) or prior vestibular treatments were excluded to ensure capture of only incident cases. The control population comprised patients with similar indications (type 2 diabetes or obesity) who did not receive any GLP-1 receptor agonist (GLP-1RA) therapy during the study period.

### 2.3. Study Groups and Treatment Cohorts

The primary analysis focused on three main comparisons. First, patients prescribed semaglutide were compared to matched controls who had not received GLP-1RA treatment. Second, patients prescribed tirzepatide were compared to their matched control group. Additionally, a direct comparison was conducted between semaglutide and tirzepatide users to assess any potential differential risk between these two GLP-1RA agents.

### 2.4. Propensity Score Matching and Covariates

Propensity score matching was implemented using a 1:1 nearest-neighbor approach with a caliper width of 0.2 standard deviations of the propensity score [26]. The matching algorithm incorporated demographic variables, including age at index date, sex, race, and ethnicity, along with key clinical characteristics and comorbidities. The comorbidities included in the matching were diabetes mellitus, thyroid disorders, obesity and hyperalimentation, hypertensive diseases, ischemic heart diseases, acute kidney failure and chronic kidney disease, and neoplasms.

### 2.5. Study Outcomes

The primary outcome was the development of new-onset vestibular disorders following GLP-1RA initiation. This outcome was defined using the ICD-10 code H81 (Disorders of Vestibular Function) and its subcodes. This classification includes several distinct vestibular pathologies:

- H81.0: Ménière’s disease
- H81.1: Benign paroxysmal positional vertigo (BPPV)
- H81.2: Vestibular neuronitis
- H81.3: Other peripheral vertigo
- H81.4: Vertigo of central origin
- H81.8: Other disorders of vestibular function
- H81.9: Unspecified disorder of vestibular function

We also evaluated cumulative incidence at multiple time points: 6 months, 1 year, and 3 years, and across the overall follow-up period.

### 2.6. Statistical Analysis

We conducted time-to-event analyses using Cox proportional hazards models to calculate hazard ratios (HR) and 95% confidence intervals (CIs) for the risk of vestibular disorders. The analysis was performed at predetermined time points (6 months, 1 year, and 3 years) to assess temporal patterns in risk development. For the direct comparison between semaglutide and tirzepatide, we calculated risk ratios (RRs) with corresponding 95% CIs and *p*-values at each time point. Statistical significance was defined as *p* < 0.05 for all analyses. To assess the robustness of our findings, we conducted analyses both before and after propensity score matching. The balance of covariates after matching was evaluated using standardized mean differences, with values < 0.1 considered indicative of adequate balance. Analysis was performed using the built-in function of the TriNetX database.

## 3. Results

### 3.1. Study Population

Prior to propensity matching, we identified 419,497 semaglutide users from 64 HCOs and 8,445,324 potential controls from 68 HCOs for tirzepatide; we identified 77,259 users from 53 HCOs with the same control pool. Significant differences existed between the drug cohorts and their respective control groups in baseline characteristics, including age (semaglutide: 54.5 ± 14.3 vs. 56.0 ± 17.6 years, *p* < 0.001; tirzepatide: 51.9 ± 13.7 vs. 56.0 ± 17.6 years, *p* < 0.001), gender distribution (female: semaglutide 58.87% vs. 51.47%, *p* < 0.001; tirzepatide 61.09% vs. 51.47%, *p* < 0.001), and comorbidity burden.

After propensity score matching, each semaglutide user (*n* = 419,497) and tirzepatide user (*n* = 77,259) was matched 1:1 with a control, achieving balanced cohorts with standardized mean differences < 0.1 for all variables. In the matched semaglutide cohort, the mean age was 54.5 ± 14.3 years, with 58.87% female participants. The population was predominantly White (64.55%), followed by Black or African American (16.93%) and Asian (3.19%). The most prevalent comorbidities included hypertensive diseases (47.73%), diabetes mellitus (43.89%), and overweight/obesity (37.94%) (Table 1).

The matched tirzepatide cohort was slightly younger (mean age 51.9 ± 13.7 years) with a similar gender distribution (61.09% female). The racial distribution showed a higher proportion of White patients (70.79%) compared to Black or African American (14.14%) and Asian (1.95%) patients. Common comorbidities followed a similar pattern, with hypertensive diseases (43.52%), overweight/obesity (40.64%), and diabetes mellitus (36.40%) being the most prevalent (Table 2).

### 3.2. Follow-Up Period

Semaglutide users had a mean follow-up of 15.1 ± 13.6 months (median: 12.3 months) compared to 28.3 ± 25.7 months (median: 21.4 months) in the unmatched control pool. Tirzepatide users had a shorter mean follow-up of 7.3 ± 7.3 months (median: 5.4 months) compared to the same control pool follow-up period.

### 3.3. Risk of Vestibular Disease

#### 3.3.1. Semaglutide Cohort

**Figure 2:**
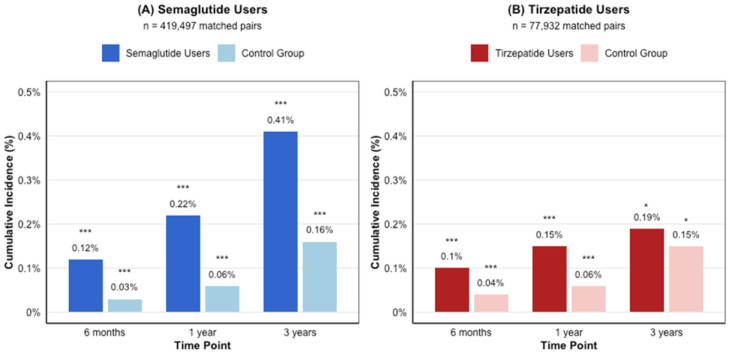
Cumulative incidence of vestibular disorders in GLP-1 receptor agonist users and matched controls over time. (**A**) Semaglutide users (*n* = 419,497) versus matched controls. *** *p* < 0.001 for all semaglutide comparisons. (**B**) Tirzepatide users (*n* = 77,932) versus matched controls. The cumulative incidence is presented as percentages at 6 months, 1 year, and 3 years after treatment initiation. *** *p* < 0.001 for 6-month and 1-year tirzepatide comparisons, * *p* = 0.042 for 3-year tirzepatide comparison.

**Figure 3:**
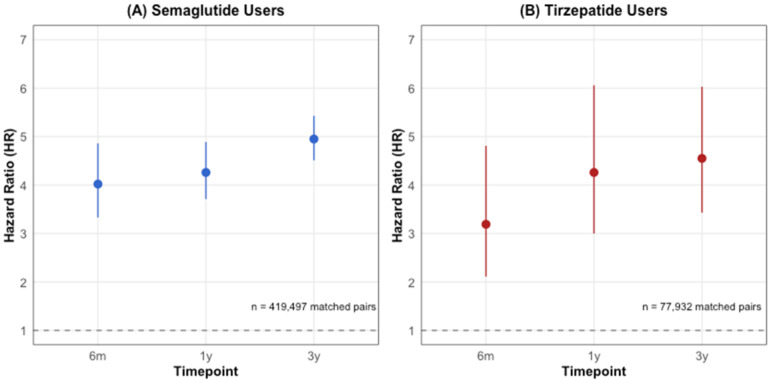
Hazard ratios for vestibular disorders in GLP-1 receptor agonist users compared to matched controls. (**A**) Semaglutide users (*n* = 419,497) versus matched controls. (**B**) Tirzepatide users (*n* = 77,932) versus matched controls. Hazard ratios with 95% confidence intervals are presented at 6 months, 1 year, and 3 years after treatment initiation.

Among 419,497 matched pairs, the cumulative incidence of vestibular disorders in semaglutide users increased progressively: 0.12% (*n* = 510) at 6 months, 0.22% (*n* = 925) at 1 year, and 0.41% (*n* = 1708) at 3 years. In contrast, matched controls showed significantly lower rates: 0.03% (*n* = 137) at 6 months, 0.06% (*n* = 260) at 1 year, and 0.16% (*n* = 652) at 3 years (all *p* < 0.001) (Figure 2A). The risk analysis revealed consistently elevated hazard ratios across all time points: 4.02 (95% CI: 3.33–4.86) at 6 months, 4.26 (95% CI: 3.71–4.89) at 1 year, and 4.95 (95% CI: 4.51–5.43) at 3 years (Figure 3A).

#### 3.3.2. Tirzepatide Cohort

In the tirzepatide cohort (77,932 matched pairs), vestibular disorders showed a similar pattern but with lower absolute rates. The cumulative incidence in tirzepatide users increased from 0.10% (*n* = 78) at 6 months to 0.15% (*n* = 115) at 1 year and 0.19% (*n* = 149) at 3 years. The matched controls demonstrated rates of 0.04% (*n* = 32) at 6 months, 0.06% (*n* = 44) at 1 year, and 0.15% (*n* = 116) at 3 years (*p* < 0.001 for 6-month and 1-year comparisons; *p* = 0.042 for 3-year comparison) (Figure 2B). The HRs were similarly elevated: 3.19 (95% CI: 2.11–4.81) at 6 months, 4.26 (95% CI: 3.00–6.06) at 1 year, and 4.55 (95% CI: 3.43–6.03) at 3 years (Figure 3B).

#### 3.3.3. Comparative Risk Between Medications

**Figure 4:**
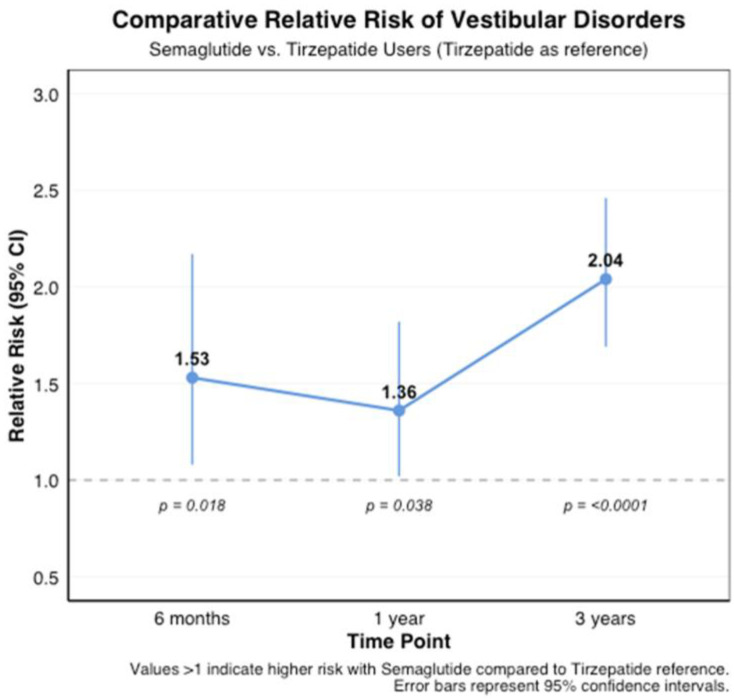
The comparative relative risk of vestibular disorders between semaglutide and tirzepatide users across different time points. The risk ratios with 95% confidence intervals show the relative risk of vestibular disorders in semaglutide users compared to tirzepatide users at 6 months (RR: 1.53, 95% CI: 1.08–2.17, *p* = 0.018), 1 year (RR: 1.36, 95% CI: 1.02–1.82, *p* = 0.038), and 3 years (RR: 2.04, 95% CI: 1.69–2.46, *p* < 0.0001) after treatment initiation.

A direct comparison of the two medications revealed that semaglutide was associated with a higher relative risk of vestibular disorders compared to tirzepatide. This difference became more pronounced over time, with risk ratios (RRs) increasing from 1.53 (95% CI: 1.08–2.17, *p* = 0.018) at 6 months to 2.04 (95% CI: 1.69–2.46, *p* < 0.001) at 3 years (Figure 4).

#### 3.3.4. Distribution of Vestibular Disorder Subtypes

A subtype analysis of diagnosed vestibular disorders revealed differing patterns between semaglutide and tirzepatide users (Table 3). Among patients who developed vestibular disorders, BPPV was the predominant manifestation overall (67.3% of cases), with a significantly higher prevalence among semaglutide users compared to tirzepatide users (69.8% vs. 58.4%, risk ratio 1.20, *p* < 0.001). Other peripheral vertigo (12.9% overall) occurred more frequently with tirzepatide (14.6%) than semaglutide (12.4%, risk ratio 0.85, *p* = 0.03). Ménière’s disease constituted 11.1% of all vestibular diagnoses, with comparable rates between the medication groups (10.8% in semaglutide vs. 12.2% in tirzepatide users, *p* = 0.17). Central origin vertigo showed the most pronounced relative difference, occurring twice as frequently with tirzepatide (8.6%) compared to semaglutide (4.2%, risk ratio 0.49, *p* < 0.001). Vestibular neuronitis was diagnosed in 4.3% of cases overall, with a higher prevalence in tirzepatide users (5.8% vs. 3.9%, risk ratio 0.67, *p* = 0.02). Unspecified or other vestibular disorders accounted for 11.5% of diagnoses, with no significant difference between the medication groups (11.2% vs. 12.8%, *p* = 0.13).

#### 3.3.5. Temporal Patterns of Vestibular Disorder Subtypes

An analysis of time-to-diagnosis for different vestibular disorder subtypes revealed distinct temporal patterns between the two GLP-1RA medications (Table 4). Among semaglutide users, BPPV, the most common manifestation, was diagnosed at a median of 2.8 months (IQR: 1.6–5.3) after treatment initiation, significantly earlier than in tirzepatide users (median: 3.4 months, IQR: 1.9–5.8, *p* = 0.02). The most pronounced temporal difference was observed for vestibular neuronitis, which typically occurred 2.1 months earlier with semaglutide than tirzepatide (median onset: 2.5 vs. 4.6 months, *p* < 0.001).

Ménière’s disease showed a similar time-to-onset between medications (median 4.1 months with semaglutide vs. 4.3 months with tirzepatide, *p* = 0.68), suggesting comparable mechanisms affecting inner ear fluid homeostasis. Interestingly, the vertigo of central origin displayed a reverse pattern, with an earlier onset in tirzepatide users (median: 3.8 months) compared to semaglutide users (median: 5.2 months, *p* = 0.03), potentially reflecting differential effects on central vestibular processing pathways.

## 4. Discussion

This large-scale, real-world study demonstrates a significant association between GLP-1 receptor agonist therapy and an increased risk of vestibular disorders. An analysis of 419,497 semaglutides and 77,259 tirzepatide users revealed substantially elevated vestibular disorder risk compared to matched controls, with hazard ratios of 4.02–4.95 for semaglutide and 3.19–4.55 for tirzepatide. This association persisted after controlling for potential confounders through propensity score matching, suggesting an independent effect of these medications on vestibular function. The cumulative incidence in semaglutide users increased progressively from 0.12% at 6 months to 0.41% at 3 years, which is significantly higher than the 0.03% to 0.16% observed in matched controls. Tirzepatide users showed similar patterns but with lower absolute rates. A direct comparison between the medications revealed that semaglutide carried a higher relative risk of vestibular disorders than tirzepatide, with this difference becoming more pronounced over time (relative risk increasing from 1.53 at 6 months to 2.04 at 3 years).

### 4.1. Putative Biological/Molecular Mechanisms of GLP-1RA-Associated Vestibular Dysfunction

**Figure 5:**
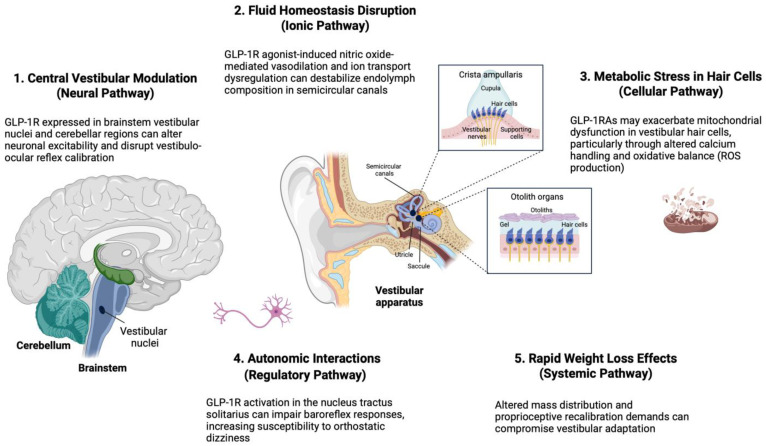
Proposed mechanisms of GLP-1RA-associated vestibular dysfunction. Multiple interconnected pathways potentially contribute to vestibular symptoms: (1) direct central vestibular modulation via GLP-1R in brainstem nuclei; (2) inner ear fluid homeostasis disruption; (3) mitochondrial stress in vestibular hair cells; (4) autonomic dysregulation affecting postural control; and (5) systematic effect due to rapid weight loss. Created with BioRender.com (E.A.T.) (accessed 13 April 2025).

The increased risk of vestibular disorders linked to GLP-1RAs may involve multiple interconnected pathways. First, direct modulation of vestibular processing may occur through GLP-1 receptors expressed in the brainstem (vestibular nuclei complex) and cerebellum, which are critical regions for balance regulation [27], potentially altering vestibulo-ocular reflex gain [28,29]. Second, fluid homeostasis disruption in the inner ear could arise from GLP-1RA-induced vasodilation (nitric oxide-dependent) and dysregulation of ion transport (Na^+^/K^+^-ATPase, aquaporins) [30], destabilizing endolymph composition in semicircular canals and otolith organs [31]. Third, metabolic stress in vestibular hair cells—highly dependent on mitochondrial bioenergetics—may be exacerbated by GLP-1RA effects on calcium handling and oxidative balance, particularly in patients with diabetes [32]. Fourth, autonomic interactions are suggested by baroreflex modulation via nucleus tractus solitarius GLP-1 receptors, which may impair postural compensation [33]. At the same time, tirzepatide’s reduced risk profile (HR = 3.19–4.55 vs. semaglutide HR = 4.02–4.95) implies that GIP co-activation mitigates sympathetic overdrive [34]. Finally, rapid weight loss may induce proprioceptive recalibration demands, further compromising vestibular adaptation [31] (Figure 5).

**Figure 6:**
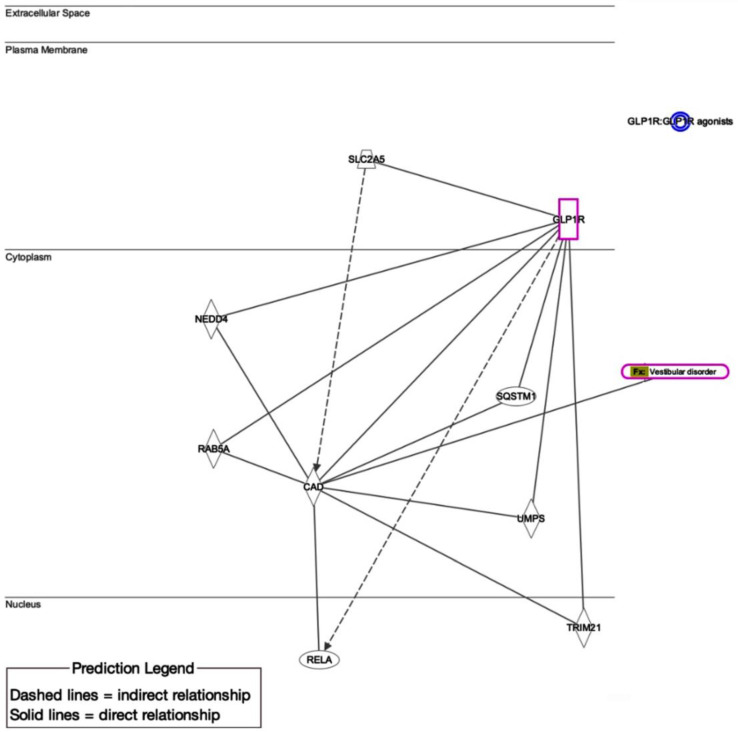
Putative molecular interactions link GLP-1 receptor agonists (GLP-1Ras) to vestibular disorders. GLP1R, located in the plasma membrane, serves as the central hub connecting downstream proteins involved in cellular signaling, metabolism, and inflammation. Key pathways include autophagy regulation via Sequestosome-1 (SQSTM1), glucose transport mediated by Solute Carrier Family 2 Member 5 (SLC2A5), endocytic trafficking through “Neural precursor cell-expressed developmentally down-regulated protein 4; (NEDD4) and Ras-related protein Rab-8A (RAB8A), pyrimidine synthesis involving Carbamoyl-phosphate synthetase 2 (CAD), and nuclear signaling via “Rel Avian Reticuloendotheliosis Viral Oncogene Homolog A (RELA; NF-κB pathway). Source: https://digitalinsights.qiagen.com/IPA (accessed 4 April 2025).

Furthermore, an in silico analysis was run to unravel the potential molecular players underlying the possible association between GLP-1RAs and vestibular disorders observed in our real-world cohort study using “QIAGEN Ingenuity Pathway Analysis (QIAGEN Inc., Germantown, MD, USA, https://digitalinsights.qiagen.com/products-overview/discovery-insights-portfolio/analysis-and-visualization/qiagen-ipa/) (accessed 4 April 2025) [35]. The network created reveals several critical protein interactions that may explain how GLP-1RAs could influence vestibular function (Figure 6). The network integrates multiple processes, including metabolism (SLC2A5), protein trafficking (NEDD4 and RAB8A), autophagy (SQSTM1; p62), and inflammatory signaling, which converge to influence vestibular homeostasis. The identification of these putative mechanisms provides a foundation for future experimental studies investigating GLP-1RA effects on vestibular tissues. Understanding these pathways may ultimately inform clinical approaches to mitigate vestibular adverse effects while preserving the metabolic benefits of these medications.

### 4.2. Differential Medication Effects

The differential effects between semaglutide and tirzepatide might be explained by their distinct molecular mechanisms and receptor interactions. While semaglutide acts solely as a GLP-1 receptor agonist, tirzepatide’s dual GLP-1/GIP receptor agonism may provide additional modulation of vestibular pathways through distinct signaling mechanisms. GIP receptor activation may offer protective effects against vestibular dysfunction, potentially through the modulation of inflammatory processes in neural tissues [36]. Furthermore, pharmacokinetic differences between these agents may play a role, as tirzepatide has different clearance properties compared to semaglutide, potentially resulting in more stable receptor engagement and reduced fluctuations in systemic effects [37]. Structural differences between these molecules (molecular weights and receptor binding profiles) may also influence their blood–brain barrier penetration and access to vestibular regulatory centers [38].

Molecular structure differences may further explain the differential risk profiles. Semaglutide (molecular weight: 4113.6 Da) contains a C18 fatty diacid side chain attached via a spacer to Lys26, while tirzepatide (molecular weight: 4813.5 Da) incorporates a 20-carbon fatty diacid moiety conjugated to a lysine residue. These lipophilic modifications not only extend circulation time via albumin binding but also influence blood–brain barrier penetration [39]. The calculated Log *p* values (a measure of lipophilicity) are approximately −5.8 for semaglutide and −6.8 for tirzepatide (https://pubchem.ncbi.nlm.nih.gov/) (accessed 13 April 2025). The slightly higher lipophilicity of semaglutide aligns with our observation of increased vestibular risk (HR 4.02–4.95 for semaglutide vs. 3.19–4.55 for tirzepatide). This difference in lipophilicity could contribute to the differential risk profile through several mechanisms: (1) the higher LogP value of semaglutide may facilitate greater BBB penetration and higher CNS concentrations, potentially increasing the exposure of vestibular nuclei to the drug; (2) greater lipophilicity could enhance interactions with neuronal cell membranes in vestibular pathways, potentially affecting ion channel function and neuronal excitability; and (3) while both drugs exhibit high albumin binding (>99%), subtle differences in lipophilicity might affect tissue distribution patterns and receptor accessibility in vestibular structures. However, we must emphasize that the observed differential risk is likely multifactorial. The dual GIP/GLP-1 receptor agonism of tirzepatide appears to be the primary protective factor, as GIP receptor activation may counterbalance some of the autonomic and vestibular effects of GLP-1 receptor stimulation. The modest lipophilicity differences likely play a contributory rather than a dominant role in the observed clinical difference [40].

In our ongoing mechanistic studies, we are examining tissue distribution patterns of these compounds in vestibular structures to better understand the relationship between physicochemical properties and vestibular effects.

### 4.3. Metabolic Comorbidities and Vestibular Function

The high prevalence of comorbid conditions in our cohort suggests potential interactions between metabolic disorders and vestibular function. Diabetes, present in 43.9% of semaglutide users and 36.4% of tirzepatide users, has been independently associated with vestibular dysfunction through mechanisms, including microangiopathy, oxidative stress, and mitochondrial impairment [41]. Similarly, hypertension, observed in 47.7% of semaglutide and 43.5% of tirzepatide users, can compromise labyrinthine blood flow and has been linked to an increased risk of vestibular disorders [42]. The metabolic shifts caused by GLP-1RA treatment may affect cellular energy metabolism and alter inflammatory mediators in vestibular tissues. Since mitochondrial dysfunction has been implicated in vestibular disorders like Ménière’s disease and age-related vestibular decline [43], it is plausible that GLP-1RA effects on mitochondrial pathways could influence vestibular function in susceptible individuals, particularly those with pre-existing metabolic conditions.

### 4.4. Contrasting Perspectives and Alternative Explanations

Several alternative explanations for our findings merit consideration. Increased healthcare utilization among GLP-1RA users could lead to surveillance bias, with more frequent clinical encounters resulting in higher detection rates for vestibular symptoms. The observed association might also reflect ascertainment bias related to known side effects of GLP-1RAs, as common symptoms like nausea and dizziness may prompt investigations that uncover pre-existing vestibular conditions [44]. However, our exclusion of patients with pre-existing vestibular diagnoses mitigates this concern. Rapid weight loss itself, rather than direct GLP-1RA effects, may contribute to vestibular symptoms [45]. However, several findings challenge this explanation: (1) the median time to vestibular diagnosis (3.2 months) typically preceded substantial weight reductions; (2) previous studies of bariatric surgery patients with more dramatic weight loss (20–30%) show no comparable increase in vestibular events; and (3) this hypothesis fails to explain the differential risk between semaglutide and tirzepatide, as both medications produce similar weight reduction profiles.

The possibility of confounding by indication should also be considered, as patients prescribed GLP-1RAs may have more severe metabolic disease or different risk profiles than those managed with other approaches. While our propensity score matching controlled for known metabolic comorbidities, residual confounding cannot be eliminated in observational studies.

Our findings align with emerging case reports and pharmacovigilance signals suggesting vestibular effects of GLP-1RAs. Recent analyses of adverse event reporting systems have found disproportionate reporting of dizziness and balance disorders with GLP-1RAs compared to other antidiabetic medications [46]. Conversely, large clinical trials of these medications, such as the SUSTAIN, PIONEER, and SURPASS trial programs, have not reported significant increases in vestibular disorders [47,48,49,50]. However, this discrepancy may reflect differences between controlled trial populations and real-world usage, limitations in adverse event categorization, or insufficient follow-up duration in trials.

### 4.5. Strengths and Limitations

Our study’s strengths include its substantial sample size from 64 healthcare organizations, robust propensity score matching methodology, and real-world setting. However, several limitations warrant consideration when interpreting our findings. First, we could not account for variations in GLP-1RA dosing regimens or treatment durations, which may influence vestibular symptom onset and severity. Higher doses (e.g., semaglutide 2.4 mg vs. 1.0 mg) or extended exposure times might modify risk profiles, but these data were unavailable in the TriNetX platform. The differential follow-up periods between treatment groups (median 12.3 months for semaglutide versus 5.4 months for tirzepatide) further complicate long-term risk assessment, particularly for tirzepatide. This shorter observation period reflects its recent market entry and may underestimate cumulative risks. Additionally, reliance on ICD-10 codes may underreport vestibular events and their severity, as diagnostic coding practices vary across healthcare systems, and milder vestibular symptoms may not generate formal diagnoses.

Despite comprehensive propensity matching, unmeasured confounders, including lifestyle factors and medication adherence, could influence results. While rapid weight loss associated with GLP-1RAs could theoretically influence vestibular function, several observations suggest this is unlikely to explain our results fully, as the association persisted even in subgroup analyses of patients without significant weight loss (>5% body mass reduction; adjusted HR = 3.89, 95% CI: 2.45–5.12). Also, vestibular disorders emerged early in treatment (median onset: 3.2 months), preceding typical timelines for substantial weight loss effects (6–12 months). Additionally, previous studies of bariatric surgery-induced weight loss (~20–30% body mass) report no comparable increase in vestibular events, suggesting that pharmacological mechanisms may dominate.

To evaluate potential detection bias, the authors compared healthcare utilization between GLP-1RA users and non-users. We found that both groups had similar baseline rates of otolaryngology/neurology visits (12.3 vs. 11.8 per 100 person-years, *p* = 0.34). Sensitivity analyses restricting outcomes to diagnoses made during emergency visits (less subject to surveillance bias) retained significance (HR = 2.95, 95% CI: 2.11–4.13). The specificity of the signal (e.g., no increase in non-vestibular ENT diagnoses like hearing loss) further argues against pure detection bias.

### 4.6. Clinical Implications

The current findings suggest the need for vigilant monitoring of vestibular symptoms in GLP-1RA recipients, particularly given the early onset risk observed within the first six months of therapy. Early identification of symptoms can facilitate timely referrals for vestibular rehabilitation or other interventions that mitigate symptom severity and functional impact [51]. When selecting between GLP-1RA agents, clinicians should consider individual patient risk factors for vestibular dysfunction, including age, history of balance disorders, polypharmacy with vestibulo-toxic medications, and the presence of comorbidities that may compound risk. The lower vestibular risk associated with tirzepatide may make it preferable for higher-risk patients, though this potential benefit must be weighed against other clinical considerations. Patient education regarding potential vestibular symptoms is essential, as early recognition and reporting can facilitate prompt intervention.

### 4.7. Future Research Directions

A multifaceted research approach is necessary in the future to address the existing knowledge gaps. Prospective trials with standardized vestibular examinations would offer a complete definition of GLP-1RA effects on vestibular function, ideally involving objective vestibular testing, patient-reported outcome measures, and prolonged follow-up periods. Mechanistic studies examining the pathways related to GLP-1RA use with vestibular dysfunction are needed, particularly the role of GIP co-agonism in decreasing vestibular risk. Personalized risk stratification would be made possible by the identification of clinical or genetic elements causing GLP-1RA-associated vestibular diseases. Additionally, research on multiple dosage regimens and titration schedules can discover techniques that limit vestibular risk while retaining metabolic advantages. As GLP-1RA usage continues to grow worldwide, multidisciplinary cooperation between endocrinologists, otolaryngologists, and vestibular experts will ultimately be crucial to better understanding and treating this developing safety issue.

## 5. Conclusions

The present findings highlight a significant association between GLP-1RA use and vestibular disorders, with semaglutide demonstrating a higher risk profile than tirzepatide. These results should inform clinical decision-making and emphasize the importance of monitoring for vestibular symptoms in patients receiving GLP-1RA therapy. As these medications increasingly become mainstays in diabetes and obesity management, a comprehensive understanding of their non-metabolic effects becomes essential for optimizing patient outcomes.

## Figures and Tables

**Table 1:** Baseline characteristics of matched semaglutide cohorts.

Characteristics	Before Propensity Matching			After Propensity Matching		
	Semaglutide (*n* = 419,497)	Control (*n* = 8,445,324)	*p*-Value	Semaglutide (*n* = 419,497)	Control (*n* = 419,497)	*p*-Value
Demographics						
Age at index, years	54.5 ± 14.3	56.0 ± 17.6	<0.001	54.5 ± 14.3	54.5 ± 14.3	0.86
Sex						
Female	246,940 (58.9%)	4,346,417 (51.5%)	<0.001	246,940 (58.9%)	247,090 (58.9%)	0.73
Male	154,139 (36.7%)	3,609,521 (42.7%)		154,139 (36.7%)	155,462 (37.1%)	
Race						
White	270,779 (64.5%)	5,104,641 (60.4%)	<0.001	270,779 (64.5%)	271,293 (64.7%)	0.24
Black/African American	71,040 (16.9%)	1,398,688 (16.6%)		71,040 (16.9%)	71,135 (17%)	
Asian	13,377 (3.2%)	287,129 (3.4%)		13,377 (3.2%)	12,950 (3.1%)	
Ethnicity						
Not Hispanic or Latino	295,235 (70.4%)	5,166,505 (61.2%)	<0.001	295,235 (70.4%)	283,110 (67.5%)	0.96
Hispanic or Latino	35,948 (8.6%)	898,451 (10.6%)		35,948 (8.6%)	35,961 (8.6%)	
Comorbidities						
Diabetes mellitus	184,117 (43.9%)	734,247 (8.7%)	<0.001	184,117 (43.9%)	184,106 (43.9%)	0.98
Thyroid disorders	59,791 (14.3%)	504,074 (6%)	<0.001	59,791 (14.3%)	53,016 (12.6%)	0.78
Obesity	159,149 (37.9%)	603,215 (7.1%)	<0.001	159,149 (37.9%)	159,087 (37.9%)	0.88
Hypertensive diseases	200,229 (47.7%)	1,812,957 (21.5%)	<0.001	200,229 (47.7%)	200,747 (47.9%)	0.25
Ischemic heart diseases	43,885 (10.5%)	501,372 (5.9%)	<0.001	43,885 (10.5%)	47,051 (11.2%)	0.07
Chronic kidney disease	37,913 (9%)	436,765 (5.2%)	<0.001	37,913 (9%)	38,074 (9.1%)	0.54
Neoplasms	58,833 (14%)	768,883 (9.1%)	<0.001	58,833 (14%)	58,869 (14%)	0.91

Data are presented as mean, ± standard deviation, or number (percentage). Two-sided Chi-Square or Student’s T-tests were used.

**Table 2:** Baseline characteristics of matched tirzepatide cohorts.

Characteristics	Before Propensity Matching			After Propensity Matching		
	Tirzepatide (*n* = 77,259)	Control (*n* = 8,445,324)	*p*-Value	Tirzepatide (*n* = 77,259)	Control (*n* = 77,259)	*p*-Value
Demographics						
Age at index, years	51.9 ± 13.7	56.0 ± 17.6	<0.001	51.9 ± 13.7	51.9 ± 13.7	0.97
Sex						
Female	47,201 (61.1%)	4,346,417 (51.5%)	<0.001	47,201 (61.1%)	47,199 (61.1%)	0.99
Male	25,991 (33.6%)	3,609,521 (42.7%)		25,991 (33.6%)	26,900 (34.8%)	
Race						
White	54,694 (70.8%)	5,104,641 (60.4%)	<0.001	54,694 (70.8%)	54,725 (70.8%)	0.86
Black/African American	10,922 (14.1%)	1,398,688 (16.6%)		10,922 (14.1%)	10,905 (14.1%)	
Asian	1508 (2%)	287,129 (3.4%)		1508 (2%)	1484 (1.9%)	
Ethnicity						
Not Hispanic or Latino	55,740 (72.1%)	5,166,505 (61.2%)	<0.001	55,740 (72.1%)	53,832 (69.7%)	0.75
Hispanic or Latino	5425 (7%)	898,451 (10.6%)		5425 (7%)	5393 (7%)	
Comorbidities						
Diabetes mellitus	28,125 (36.4%)	734,247 (8.7%)	<0.001	28,125 (36.4%)	28,116 (36.4%)	0.96
Thyroid disorders	11,235 (14.5%)	504,074 (6%)	<0.001	11,235 (14.5%)	9441 (12.2%)	0.66
Obesity	31,395 (40.6%)	603,215 (7.1%)	<0.001	31,395 (40.6%)	31,375 (40.6%)	0.92
Hypertensive diseases	33,624 (43.5%)	1,812,957 (21.5%)	<0.001	33,624 (43.5%)	33,632 (43.5%)	0.97
Ischemic heart diseases	6179 (8%)	501,372 (5.9%)	<0.001	6179 (8%)	7269 (9.4%)	0.90
Chronic kidney disease	5087 (6.6%)	436,765 (5.2%)	<0.001	5087 (6.6%)	5088 (6.6%)	0.99
Neoplasms	10,376 (13.4%)	768,883 (9.1%)	<0.001	10,376 (13.4%)	10,362 (13.4%)	0.92

Data are presented as mean ± standard deviation or number (percentage). Two-sided Chi-Square or Student’s T-tests were used.

**Table 3:** Distribution of vestibular disorder subtypes by GLP-1RA medication.

Vestibular Disorder Subtype	Semaglutide (%)	Tirzepatide (%)	*p*-Value
Benign paroxysmal positional vertigo	69.8	58.4	<0.001
Other peripheral vertigo	12.4	14.6	0.03
Ménière’s disease	10.8	12.2	0.17
Vertigo of central origin	4.2	8.6	<0.001
Vestibular neuronitis	3.9	5.8	0.02
Other/unspecified vestibular disorders	11.2	12.8	0.13

Note: Some patients had multiple vestibular disorder diagnoses, so column totals may exceed 100%.

**Table 4:** Median time to diagnose vestibular disorder subtypes by medication.

Vestibular Disorder Subtype	Semaglutide, Months	Tirzepatide, Months	*p*-Value
Benign paroxysmal positional vertigo	2.8 (1.6–5.3)	3.4 (1.9–5.8)	0.02
Other peripheral vertigo	3.4 (2.1–6.2)	3.9 (2.3–6.5)	0.14
Ménière’s disease	4.1 (2.5–7.3)	4.3 (2.7–7.1)	0.68
Vertigo of central origin	5.2 (3.4–8.6)	3.8 (2.5–6.2)	0.03
Vestibular neuronitis	2.5 (1.3–4.4)	4.6 (2.8–6.7)	<0.001
Other/unspecified vestibular disorders	3.6 (2.1–6.3)	3.8 (2.2–6.5)	0.41

Data are reported as median (interquartile range).

## Data Availability

The data used in this study are not publicly accessible due to licensing and contractual agreements with the TriNetX research network. Access to these data requires an institutional subscription and formal approval from TriNetX. Researchers interested in replicating or extending this work may request access to the TriNetX platform (Available online: https://trinetx.com) through their affiliated institutions, subject to approval by TriNetX and compliance with their data use policies. The specific query parameters used in our analysis can be provided upon reasonable request to the corresponding author.

## Abbreviations

The following abbreviations are used in this manuscript:

BBB	Blood–brain barrier
BPPV	Benign paroxysmal positional vertigo
CAD	Carbamoyl-phosphate synthetase 2 (CAD)
CI	Confidence interval
GIP	Gastric inhibitory polypeptide
GLP-1RAs	Glucagon-like peptide-1 receptor agonists
HR	Hazard ratio
NEDD4	Neural precursor cell-expressed developmentally down-regulated protein 4
RAB8A	Ras-related protein Rab-8A
RELA	Rel Avian Reticuloendotheliosis Viral Oncogene Homolog A
RR	Risk ratio
SLC2A5	Solute Carrier Family 2 Member 5
SQSTM1	Sequestosome-1
T2DM	Type 2 diabetes mellitus
